# Ibrutinib as the Unifying Culprit: A Case Report of Multisystem Complications in an Elderly Patient With Low-Grade B-Cell Lymphoproliferative Disorder

**DOI:** 10.7759/cureus.115495

**Published:** 2026-08-31

**Authors:** Khalid Ghalib, Ahmed Elnour, Nada Eltigani Hassan Mahgoub, Mohamed Ali, Pedro Arregui

**Affiliations:** 1 Internal Medicine, University Hospital Kerry, Tralee, IRL; 2 Hematology, University Hospital Kerry, Tralee, IRL; 3 Geriatrics, Cork University Hospital, Cork, IRL

**Keywords:** adverse drug events, cd5-negative b-cell lymphoproliferative disorder, ibrutinib, multidisciplinary management, waldenstrom's macroglobulinaemia

## Abstract

Ibrutinib, an irreversible inhibitor of Bruton's tyrosine kinase (BTK), is a cornerstone of therapy for low-grade B-cell lymphoproliferative disorders (B-cell LPD), including Waldenstrom's macroglobulinaemia (WM). While highly effective, its adverse effect profile is broad and potentially life-threatening, particularly in elderly patients with multiple comorbidities. We report an 82-year-old woman with a seven-year history of CD5-negative low-grade B-cell LPD and IgM paraproteinaemia, managed sequentially with rituximab and ibrutinib. During ibrutinib therapy, she developed a cascade of complications, including recurrent infections, platelet dysfunction, new-onset paroxysmal atrial fibrillation (AF), and haemothorax following a fall and pericardial tamponade requiring urgent pericardiocentesis. The concurrent prescription of apixaban for AF and clarithromycin for lower respiratory tract infection (LRTI) created critical drug interaction hazards. A review of her clinical course suggests that ibrutinib may have been the unifying causative agent across multiple organ systems. This case highlights the importance of vigilant monitoring for ibrutinib-associated adverse events in elderly patients, the complexity of anticoagulation decisions in the setting of ibrutinib-induced AF, and the indispensable role of multidisciplinary team (MDT) management. Clinicians should maintain a high index of suspicion for ibrutinib as the culprit when patients on this therapy develop cardiovascular, haematological, infectious, or dermatological complications.

## Introduction

Waldenström's macroglobulinaemia (WM) is an indolent B-cell neoplasm defined by bone marrow infiltration with lymphoplasmacytic lymphoma and the presence of a monoclonal immunoglobulin M (IgM) paraprotein [1]. It accounts for approximately 1-2% of all haematological malignancies, with an annual incidence of approximately three cases per million population [2]. The disease predominantly affects older adults, with a median age at diagnosis of 71 years, and is more common in males [3]. In this report, we use the term WM/LPL to describe the patient's final clinicopathological diagnosis while acknowledging that the initial marrow immunophenotype was described more broadly as a CD5-negative low-grade B-cell lymphoproliferative disorder.

The MYD88 L265P somatic mutation is present in most cases of WM and has important pathogenic and therapeutic implications, including sensitivity to BTK inhibition [4].

Ibrutinib, a first-in-class irreversible (Bruton's tyrosine kinase BTK) inhibitor, has become an important treatment option in WM, with the iNNOVATE trial demonstrating superior outcomes for ibrutinib plus rituximab compared with rituximab alone [5]. Despite this efficacy, ibrutinib has a broad and clinically significant adverse effect profile. Recognised toxicities include bleeding related to platelet dysfunction [6], atrial fibrillation and hypertension [7,8], increased susceptibility to infection [9], musculoskeletal pain [10], and dermatological toxicity [11,12]. The management of these adverse effects is particularly challenging in elderly patients with pre-existing comorbidities and polypharmacy.

Although these adverse effects are individually well described, less attention has been given to how they may occur concurrently, interact with one another, and be amplified by prescribing cascades in routine clinical practice. In particular, the combination of bleeding tendency, arrhythmia prompting anticoagulation, infection requiring antimicrobial therapy, and exposure to CYP3A4 inhibitors may create a complex multisystem presentation in which drug-related toxicity is not immediately recognised.

We present the case of an 82-year-old woman with WM/LPL treated with ibrutinib who developed a sequence of complications including falls, haemothorax, infection, atrial fibrillation, anticoagulation complexity, and pericardial tamponade. The specific contribution of this case lies not in any single adverse event, but in illustrating how ibrutinib may have contributed to a multifactorial cascade across several organ systems in a frail older patient, thereby complicating both diagnosis and management.

## Case presentation

Patient background and initial diagnosis

An 82-year-old woman with a longstanding history of hypertension and hypercholesterolaemia presented with anaemia and an incidentally detected IgM paraprotein of 9.7 g/L (IgM band: 5 g/dL). Bone marrow examination in December 2019 showed extensive infiltration by B lymphocytes. Immunophenotyping demonstrated a clonal CD5-negative low-grade B-cell lymphoproliferative disorder, and the overall clinicopathological picture, together with the presence of an IgM paraprotein, was considered most consistent with WM/LPL (Table 1). Anti-Ro antibody positivity was concurrently identified by the rheumatology team, raising the possibility of an underlying Sjogren's syndrome or connective tissue disease overlap - a recognised predisposing condition for B-cell LPD, with primary Sjogren's syndrome carrying a 7- to 44-fold increased risk of non-Hodgkin lymphoma [13].

**Table 1:** Bone marrow aspirate immunophenotyping findings WBC, white blood cell count; Hb, haemoglobin; CD, cluster of differentiation; NHL/LPD, non-Hodgkin lymphoma/lymphoproliferative disorder; *, not reported in source record Bone marrow aspirate immunophenotyping showed no phenotypic evidence of myeloma. A clonal B-cell population comprising 30% of nucleated cells was identified, with immunophenotype consistent with CD5-negative B-cell NHL/LPD.

Section	Details
Sample type	Bone marrow aspirate sample in EDTA
Clinical details	Anaemia, high ESR, rule out myeloma
WBC	5.2 x 10^9^/L
Hb	*
Platelets	*
Neutrophils	*
Lymphocytes	*
Myeloma	No phenotypic evidence of myeloma in this aspirate
Flow cytometry	Clonal B-cell population present, representing 30% of nucleated cells
Phenotype positive	CD19+, CD20+, CD22+, CD79b+, CD200 weak+, lambda+
Phenotype negative	CD3-, CD5-, CD10-, CD23-, CD38-, CD43-, CD56-, CD103-, FMC7-, kappa-
Interpretation	Consistent with CD5-negative B-cell NHL/LPD
Advice	Correlate with clinical findings, bone marrow morphology, and other relevant investigations

Haematological course

From May to September 2020, the patient required repeated transfusions of red cell concentrates for severe anaemia, with haemoglobin ranging approximately between 6 and 8 g/dL. The anaemia was considered multifactorial, reflecting marrow infiltration, autoimmune haemolysis, iron deficiency, and chronic disease.

She received rituximab 375 mg/m² from September to December 2020. During treatment, the serum IgM increased transiently from 9.7 g/L to 16.7 g/L, consistent with an IgM flare rather than definite treatment failure [14].

By March 2022, the patient had developed pancytopenia with large bone marrow infiltration of B-cell LPD and a serum IgM of 14.7 g/L, necessitating RCC transfusions (Table 2). A second course of rituximab provided insufficient disease control. Ibrutinib 140 mg was therefore commenced in May 2022, initially on five days per week and increased to daily dosing in June 2022. Supportive therapy included weekly darbepoetin alfa from June to September 2022 and weekly granulocyte colony-stimulating factor from September 2022 to February 2023.

**Table 2:** Full blood count showing disease progression prior to ibrutinib initiation with evidence of leukopenia, anaemia, and mild thrombocytopenia WBC, white blood cell count; RBC, red blood cell count; Hb, haemoglobin; MCH, mean corpuscular haemoglobin

Test	Result	Reference range	Interpretation
WBC	2.47	4.4-11.3 x 10^9^/L	Low
RBC	2.86	3.9-5.3 x 10^12^/L	Low
Hb	9.0	11.7-15.9 g/dL	Low
MCH	31.5	26.0-34.0 pg	Normal
Platelets	134	140-440 x 10^9^/L	Low
Blood film	Examined	-	Peripheral smear reviewed

Response during ibrutinib therapy

Following ibrutinib initiation, the patient's haematological parameters improved gradually. By March 2026, the full blood count demonstrated a haemoglobin of 11.8 g/dL, white cell count of 6.7 × 10^9^/L, platelet count of 442 × 10^9^/L, and neutrophil count of 5.8 × 10^9^/L, reflecting a meaningful treatment response (Table 3). 

**Table 3:** Full blood count revealing recovery of haemoglobin, white cell count, and platelet counts as evidence of haematological improvement after prolonged ibrutinib therapy WBC, white blood cell count; RBC, red blood cell count; Hb, haemoglobin; HCT, haematocrit; MCV, mean corpuscular volume; MCH, mean corpuscular haemoglobin; MCHC, mean corpuscular haemoglobin concentration; RDW, red cell distribution width

Test	Result	Reference range	Interpretation
WBC	6.7	4.4-11.3 x 10^9^/L	Normal
RBC	4.08	3.9-5.3 x 10^12^/L	Normal
Hb	11.8	11.715.9 g/dL	Normal/lower end
HCT	0.354	0.35-0.46 L/L	Normal
MCV	86.8	80-96 fL	Normal
MCH	28.9	26.0-34.0 pg	Normal
MCHC	33.3	31.0-37.0 g/dL	Normal
RDW	15.4	11.6-15.0%	Mildly high
Platelets	442	140-440 x 10^9^/L	Mildly high
Neutrophils	5.8	1.4-6.6 x 10^9^/L	Normal
Lymphocytes	0.48	0.9-3.2 x 10^9^/L	Low
Monocytes	0.3	0.15-1.3 x 10^9^/L	Normal
Eosinophils	0.03	0.04-0.4 x 10^9^/L	Low
Basophils	0.11	0.0-0.1 x 10^9^/L	Mildly high

Major complications during prolonged ibrutinib exposure

Musculoskeletal Symptoms, Gait Instability, and Fall

Ibrutinib is associated with arthralgia in 18% and myalgia in 10% of patients, arising from off-target inhibition of tyrosine kinase expressed in hepatocellular carcinoma (TEC) and IL-2-inducible T-cell kinase (ITK) [10]. These adverse effects predominantly occur during the first year of treatment and are typically low-grade (grades 1-2 in 97-99% of cases) [10]. In this patient, musculoskeletal discomfort - in the context of pre-existing osteoarthritis and vertigo - may have contributed to impaired mobility and gait instability. In February 2026, the patient sustained a fall resulting in fractures of six left ribs and a small left-sided haemothorax, representing her first recorded fall (Figure 1).

**Figure 1:**
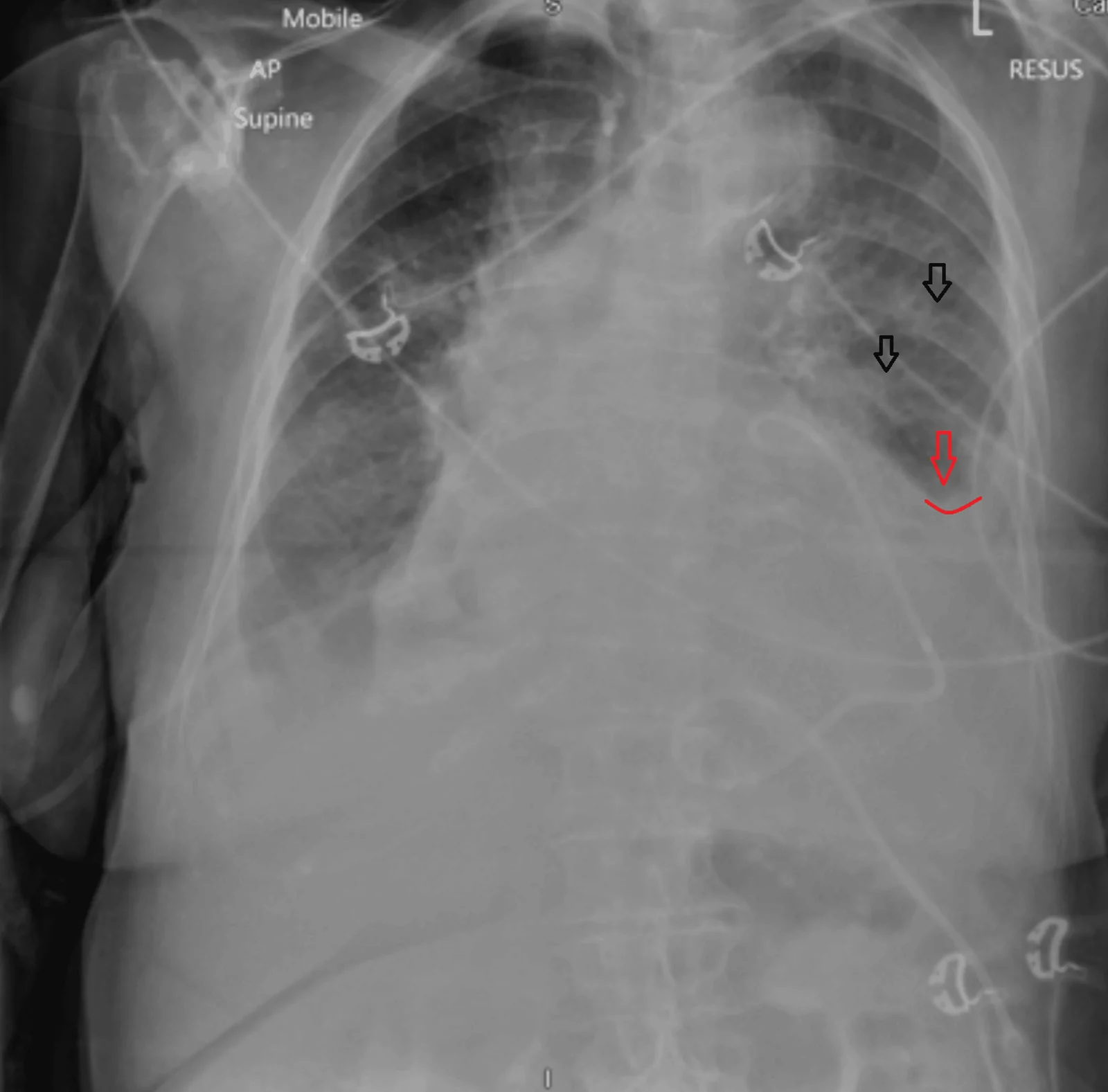
Chest radiograph following fall-related thoracic trauma Portable anteroposterior supine chest radiograph obtained following a fall, demonstrating left-sided thoracic injury with fractures of six left ribs (black arrows demonstrating cortical interruption) and a left haemothorax (red arrow and line showing blunted costophrenic angle).

Haemothorax and Platelet Dysfunction

Ibrutinib exerts potent antiplatelet effects through inhibition of Bruton tyrosine kinase (BTK)-dependent platelet signalling pathways, including the glycoprotein VI (GPVI) and C-type lectin-like receptor 2 (CLEC-2) pathways [6]. Kazianka et al. demonstrated that ristocetin-induced platelet aggregation (RIPA) values decline significantly within two weeks of ibrutinib initiation and recover within seven days of drug cessation [6]. Clinically, bleeding events of any grade occur in 44-61% of patients on ibrutinib (6). In this patient, ibrutinib-induced platelet dysfunction is likely to have amplified the haemorrhagic component of the post-traumatic haemothorax, converting what might otherwise have been a minor pleural injury into a clinically significant haemothorax requiring hospital admission.

Recurrent Lower Respiratory Tract Infection

Ibrutinib impairs BTK-dependent B-cell receptor signalling and natural killer (NK) cell function, resulting in secondary immunodeficiency that increases susceptibility to bacterial, viral, and fungal infections [9]. Ball et al. reported that, while B-cell malignancies themselves confer inherent immunosuppression, ibrutinib further compounds this risk [9]. The patient's secondary LRTI following rib fractures, treated with intravenous steroids, amoxicillin-clavulanate, and clarithromycin, is consistent with ibrutinib-associated immunosuppression. 

New-Onset Atrial Fibrillation

During her hospitalisation, the patient developed new-onset AF (Figure 2), a well-documented complication of ibrutinib therapy, with an incidence of 6-16% in clinical trials [8]. The mechanism involves on-target inhibition of BTK and the structurally related TEC kinase in cardiac tissue, leading to impairment of the PI3K-Akt signalling pathway and subsequent atrial remodelling [7]. A systematic review and meta-analysis by Caldeira et al. confirmed that ibrutinib significantly increases the risk of both AF and hypertension compared to non-ibrutinib controls [8]. Mato et al. further demonstrated that left atrial abnormality (LAA) on pre-treatment electrocardiogram is a significant predictor of ibrutinib-associated AF, with an odds ratio of 9.1 (95% CI: 2.2-37.3) [15]. This patient's longstanding hypertension and atherosclerosis represented additional predisposing factors.

**Figure 2:**
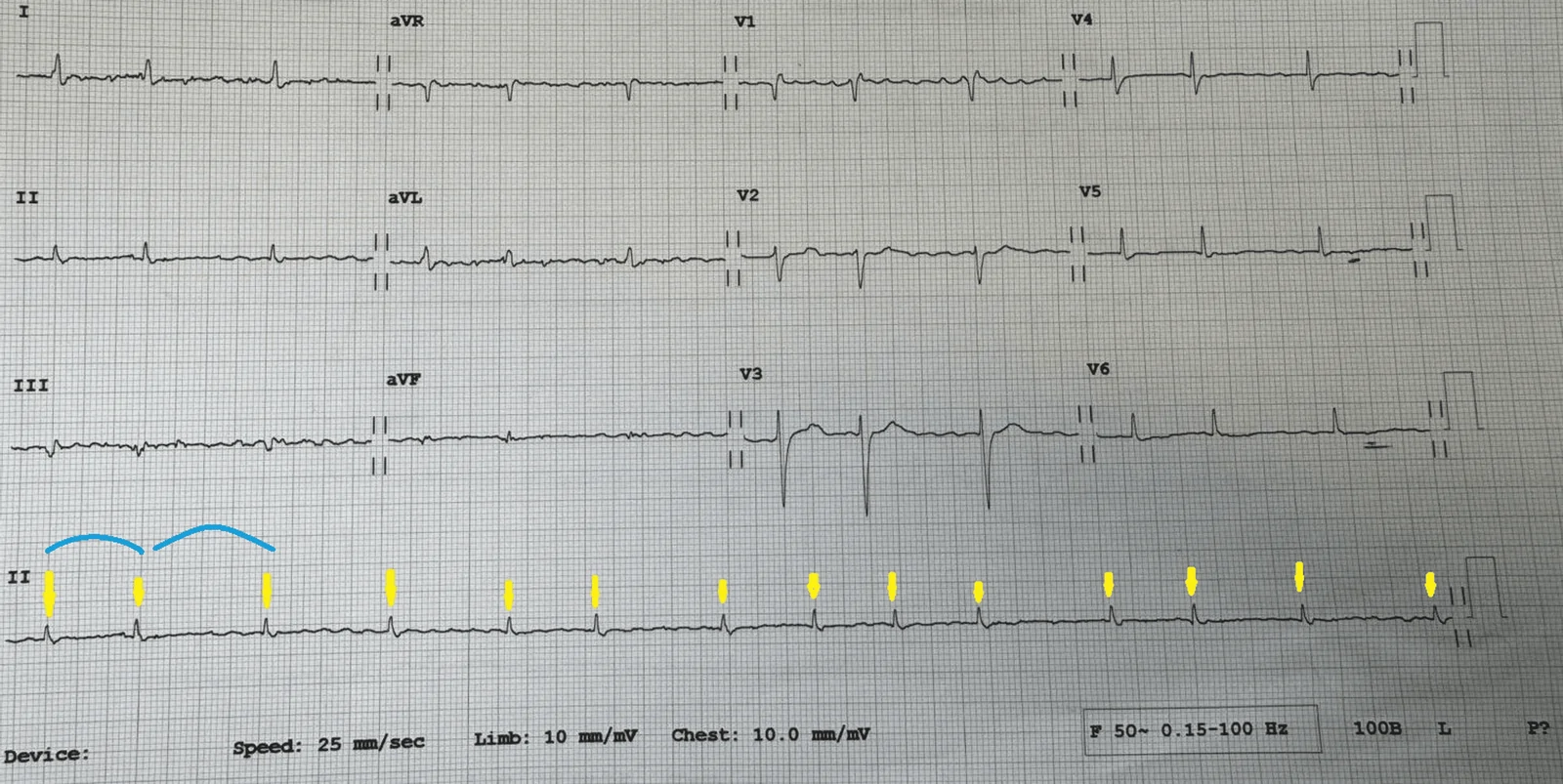
Twelve-lead electrocardiogram demonstrating atrial fibrillation with an irregularly irregular rhythm (yellow arrows) and the absence of discrete P waves

The Anticoagulation Dilemma: Ibrutinib and Apixaban

The development of AF necessitated anticoagulation to reduce thromboembolic risk. The patient's CHA₂DS₂-VASc score was high (age ≥75 years, female sex, hypertension, vascular disease), equal to 5 points, which means 7.2% stroke risk per year, clearly mandating anticoagulation. However, ibrutinib's antiplatelet effects create a substantially elevated bleeding risk when combined with anticoagulant therapy. Miatech et al. reported a case of ibrutinib-associated cardiac tamponade due to haemorrhagic pericardial effusion in a patient receiving concurrent antiplatelet therapy, illustrating the potential for catastrophic haemorrhagic complications when ibrutinib is combined with agents that further impair haemostasis [16]. Apixaban 2.5 mg twice daily (reduced dose, appropriate for age ≥80 years and weight less than 60 kg) was selected as the preferred anticoagulant, consistent with current guidance favouring direct oral anticoagulants (DOACs) over warfarin in ibrutinib-treated patients due to their lower overall bleeding risk. The HAS-BLED score was elevated (3 points), necessitating close clinical monitoring.

Critical Drug Interaction: Clarithromycin and Ibrutinib

A critical and potentially under-recognised drug interaction arose during this admission. Clarithromycin (500 mg twice daily), prescribed for LRTI, is a potent inhibitor of cytochrome P450 3A4 (CYP3A4), the principal enzyme responsible for ibrutinib metabolism. Co-administration of a strong CYP3A4 inhibitor can increase ibrutinib plasma concentrations by three to seven-fold, substantially increasing the risk of ibrutinib-associated toxicities, including bleeding, AF, and diarrhoea [17]. This interaction was present in this patient during her most complex clinical period. Switching to azithromycin, a weaker CYP3A4 inhibitor, or temporarily reducing the ibrutinib dose per the summary of product characteristics, should be considered in such circumstances.

Acute Presentation: April 2026

In April 2026, the patient was referred urgently to the emergency department with acute dyspnoea and suspected new pleural effusion, shortly after discharge from hospital in March 2026. Further evaluation identified a pericardial tamponade (Figure 3) requiring pericardiocentesis, and haematology was consulted in the acute setting. Given the markedly elevated INR of 7.1 on presentation, they advised temporary cessation of both apixaban and ibrutinib; however, reversal was not considered necessary at that time. The differential diagnosis included recurrent or residual haemothorax, reactive pleural effusion secondary to lower respiratory tract infection, WM-related pleural involvement, decompensated cardiac failure in the setting of new atrial fibrillation, and recurrent pneumonia in an immunocompromised host; pulmonary embolism was considered less likely given ongoing apixaban therapy. This presentation illustrates the cumulative burden of ibrutinib-related adverse events on the patient's clinical course.

**Figure 3:**
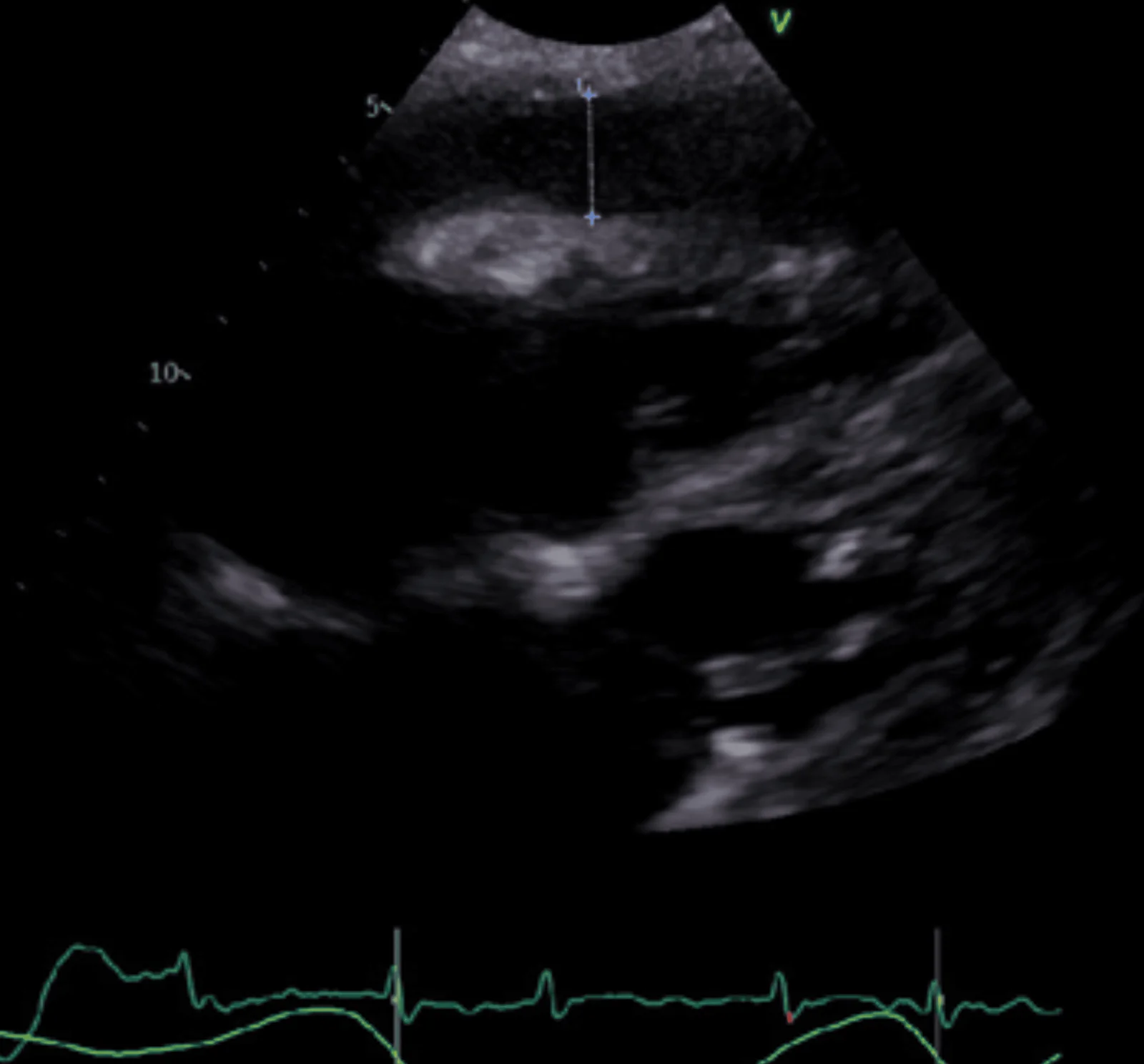
Transthoracic echocardiographic image demonstrating a pericardial effusion (white blue measurement caliper line), with fluid seen surrounding the heart and measured on the image This effusion subsequently required pericardiocentesis.

## Discussion

This case illustrates a compelling argument that ibrutinib may have been the unifying causative agent across multiple organ systems in this patient. The temporal relationship between ibrutinib commencement and the subsequent emergence of musculoskeletal symptoms, infection susceptibility, new-onset AF, platelet dysfunction, haemothorax, and pericardial tamponade is consistent with the known adverse effect profile of this drug.

The broad toxicity of ibrutinib arises from both on-target and off-target kinase inhibition. On-target BTK inhibition accounts for its anti-lymphoma efficacy but also mediates platelet dysfunction (via GPVI/CLEC-2 pathways) and cardiac arrhythmogenesis (via PI3K-Akt impairment in atrial tissue) [6,7]. Off-target inhibition of EGFR, TEC, ITK, c-Kit, and platelet-derived growth factor receptor (PDGFR) underlies musculoskeletal pain, skin toxicity, and gastrointestinal adverse effects [10-12]. Immunosuppression results from BTK-dependent impairment of B-cell and NK-cell function [9].

The UK and Ireland real-world analysis of 315 patients with relapsed/refractory CLL treated with ibrutinib demonstrated a one-year discontinuation-free survival of 73.7% and a one-year overall survival of 83.8% [17]. Importantly, dose reductions alone did not adversely affect overall survival [17], suggesting that ibrutinib dose modification, rather than discontinuation, is a reasonable strategy when managing adverse effects in frail elderly patients. Performance status emerged as the strongest predictor of outcome, reinforcing the importance of proactive frailty management alongside haematological therapy [17].

The co-existence of anti-Ro antibody positivity and Raynaud's phenomenon in this patient raises the possibility of an underlying primary Sjogren's syndrome, which itself carries a 7- to 44-fold increased risk of non-Hodgkin lymphoma, particularly B-cell subtypes [13]. It is plausible that chronic B-cell stimulation in the context of Sjogren's syndrome contributed to the development of this patient's low-grade B-cell LPD - a well-recognised association in the literature [13]. The autoimmune background may also have contributed to the positive direct antiglobulin test (DAT) and warm autoantibody formation, compounding the transfusion challenge.

The management of this patient required coordinated input from haematology, emergency medicine, respiratory medicine, cardiology, rheumatology, gastroenterology, transfusion medicine, nutrition, pharmacy, and general practice. The multidisciplinary team (MDT) model is not merely desirable in such cases; it is essential. No single specialty possesses the breadth of expertise required to navigate the competing priorities of anticoagulation, bleeding risk, infection management, haematological disease control, and frailty assessment simultaneously.

Strengths

This case highlights the challenge of distinguishing disease-related complications from treatment-related toxicity in elderly patients with lymphoproliferative disorders.

Polypharmacy and drug interactions: Polypharmacy significantly amplified the complexity of this patient's management.

This case underscores the importance of comprehensive geriatric assessment and multidisciplinary collaboration when managing older adults receiving novel targeted therapies. Early recognition of treatment-related toxicity may prevent a cascade of adverse clinical events.

Limitations

Although a causal relationship between ibrutinib and the observed complications cannot be definitively proven, the biological plausibility and temporal sequence of events provide strong supportive evidence.

## Conclusions

This case demonstrates that ibrutinib, while therapeutically crucial for the management of low-grade B-cell LPD, may function as the unifying culprit behind a cascade of multisystem complications in elderly patients. Clinicians prescribing ibrutinib must maintain vigilance for its broad adverse effect profile, encompassing cardiovascular, haematological, infectious, musculoskeletal, and dermatological domains, and should proactively screen for and manage these complications. Drug interactions, particularly with CYP3A4 inhibitors, such as clarithromycin, must be carefully considered. Anticoagulation decisions in ibrutinib-treated patients with AF require individualised risk-benefit analysis, with DOACs preferred over vitamin K antagonists. A proactive MDT approach, incorporating frailty assessment and falls prevention, is essential to optimise outcomes in this vulnerable patient population.
